# Compositionality in human and animal communication

**DOI:** 10.1007/s10071-025-02010-8

**Published:** 2025-11-06

**Authors:** Nathan Klinedinst

**Affiliations:** Psychology & Language Sciences, University College London, London, UK

**Keywords:** Animal communication, Evolution of lanugage, Animal linguistics, Compositionality, Semantics, Syntax, Pragmatics

## Abstract

Human languages use complex, structured signals whose meanings are compositional. Recent empirical research has claimed to demonstrate compositionality in bird and primate communication (Berthet et al. 2025; Engesser et al. 2016; Girard-Buttoz et al. 2025; Leroux et al. 2023; Suzuki et al. 2017). While the compositionality of human languages seems beyond doubt, it can be demonstrated powerfully and immediately because of several other important features of language. Clarifying the arguments for compositionality in human languages reveals open questions and alternative hypotheses about the evidence from other species, and directions for further research and possible limitations.

## Introduction

In human languages, the communicated signals, sentences, are formed by combining basic meaningful elements into successively larger ones (morpheme into phrases then sentences). The meanings of wholes are determined by those of the parts and how they are combined (syntax). This property is called compositionality.^1^

Recent research has claimed to establish compositionality in the natural communication systems of several non-human species (Berthet et al. 2025; Engesser et al. 2016; Girard-Buttoz et al. 2025; Leroux et al. 2023; Suzuki et al. 2017). The aim of this article is to clarify how we know that human languages are compositional (§ 2) and in doing so identify open questions about the evidence in support of compositionality in other species, and directions for future research to address them (§ 3).

## Compositionality in human languages

Compositionality may seem self-evident when it comes to human languages, but it is a hypothesis. It is directly supported by simple linguistic-behavioural facts, but may be expected to have broader psychological and neural signatures (Pylkkanen 2019). Since the essence of compositionality is use of a rule to build meaning, direct evidence takes the form of evidence of rule use. This section introduces aspects of language supporting compositionality which have been foundational in theorizing about human language (Larson 1996; Szabó 2024). The goal is not to identify every conceivable argument, but rather to clarify the nature and significance of compositionality, providing critical perspective for assessing evidence from other species (§ 3).

Why does the compositionality of human languages seem self-evident? One reason is that systematic connections between form and meaning are readily apparent. Phrases and sentences whose words are organized in the same way have perceptibly related meanings. Put another way, replacing words within a phrase or sentence changes its meaning predictably. For example: the noun phrases ‘bad dancer’, ‘bad singer’, ‘fast singer’ and ‘fast dancer’ refer to different types of people, but which type is determined in parallel fashion between cases. The adjectives (‘bad’, ‘fast’) describe a quality, and when combined with nouns built from an action verb plus the suffix -*er* (‘singer’, ‘dancer’), the resulting noun phrase can refer to people whose performance of the action has that quality. Another example comes from “action” verbs like ‘kiss’, ‘slap’, etc. In the sentences that can be formed by combining such verbs with two nouns (e.g. ‘Mary kisses Bill’, ‘Bill kisses Sue’, ‘Mary slaps Sue’, etc.), the individual named by first noun (subject) is systematically understood as performing the action and the one named by the second (direct object) as undergoing it.

This property, systematicity, makes compositionality plausible and explanatory (Szabó 2024). For example, from a cognitive perspective, considerations of efficiency of storage favor compositionality over the apparent alternative: that in addition to the meanings of individual words and syntactic rules for combining them into phrases and sentences, the meaning of *each phrase* and sentence thereby formed is stored as a unit. On the latter view, the contribution of structure to meaning is in effect stored redundantly for each related phrase and sentence. Positing instead compositional rules that yield the phrasal and sentence meanings from word meanings plus syntax not only eschews such redundancy but captures an apparent generalization. The same point can be made over and over with other types of phrases. Still, it is *logically* possible for a person to store as a unit the meanings of the four phrases ‘bad dancer’, ‘bad singer’, ‘fast singer’, ‘fast dancer’ – or, for that matter, 1000 such phrases. And practically speaking, a 1 st or 2nd language learner must likely infer at least some aspects of compositional rules and this may take time, during which memorization could be a crutch.

But three further properties of human language can be leveraged to establish compositionality directly (Larson 1996; Szabó 2024). These properties guarantee the possibility of novel phrases and sentences: ones that a speaker has never encountered previously. A fortiori the meanings of novel expressions *cannot have been memorized* as units, and spontaneous understanding of such expressions provides strong evidence for compositionality. The first property is that words, and aspects of syntax and semantics, are learned not innate. Because of this, for a person who understands e.g. ‘bad dancer’ and ‘mopper’ but has never encountered ‘bad mopper’, the latter provides a test to establish use of a (particular) compositional rule. Importantly, if ‘bad mopper’ were understood innately, the test would be inconclusive: spontaneous understanding of a novel expression would be compatible with use of a(n innate) compositional rule, but also with direct association of meaning with whole as a unit (cf. § 3 below).

The second two properties ensure that for any given speaker, a novel expression can be found *no matter the extent* of their prior experience. open-endedness refers to the fact that new words can be created and added to the language. If a new word ‘grimmer’ is coined, ‘bad grimmer’ is novel, and it is immediately clear what the expected meaning is. While acquiring a newly coined word would seem to involve the same mechanisms used to acquire pre-existing vocabulary, learning and open-endedness are logically separable: for example, a communication system could be replete with innate vocabulary yet also allow for extension by learning in principle^2^, and a learned system could be fixed and stable, without possibility of innovation.

Finally and most powerfully, recursion, or the use of self-embedding syntactic rules, yields an *infinite* class of novel expressions – even from a fixed vocabulary. For example, the prefix ‘great’ can be combined with the nouns ‘grandmother’, ‘grandfather’, ‘aunt’ and ‘uncle’ to form a noun phrase. The resulting phrase (e.g. ‘great grandmother’) can itself combine with ‘great’ forming another phrase (‘great great grandmother’) of the same type with the original one inside of it, and so on. Since memory is finite, for any speaker there is a maximal *n* such that they could have prior experience with the phrase ‘great_n_… great_1_ grandmother’, and thus ‘great_n+i_… great_1_ grandmother’ is novel for all $\:i\ge\:1$. Like the case of ‘bad mopper’ above, to use such a phrase as a “test” to demonstrate use of a compositional rule – to the effect that ‘great (great…) grandmother’ denotes one’s *parent’s* (parent’s…) grandmother – aspects of a subject’s prior experience must be known, here the value of *n*. But it is also apparent directly from introspection that we have the capacity to understand an infinity of such phrases and thus novel ones. As with open-endedness, the argument for compositionality is independent of learning in principle: whether innate or learned a list is finite, but even innate rules can be recursive.

## Compositionality in other species?

Several recent studies claim to establish compositionality in the natural communication of other species: bonobos (Berthet et al. 2025), chimpanzees (Girard-Buttoz et al. 2025; Leroux et al. 2023), Japanese great tits (Suzuki et al. 2017), and southern pied babblers (Engesser et al. 2016). These species produce calls with social and alarm functions among others. The studies propose that certain calls appear not only individually, but in pairs in close temporal sequence, in fixed order. These apparent two-call combinations are proposed to have a meaning determined or built from the component calls. For example, the Japanese great tit ABC + D call, which elicits mobbing of predators (Suzuki et al. 2017), is built from the individual calls ABC, “alert”, and D, which functions to recruit conspecifics.^3^

Schlenker et al. (2024) suggest an alternative analysis for the case of the Japanese great tit ABC + D call: as a sequence of **separate utterances**, with the ordering determined by a general cognitive principle (Schlenker et al. 2016a, b, 2023; Suzuki and Matsumoto 2022). As Schlenker et al. argue, the fact that an alert and a recruit call *come from the same source* may suffice to explain that they lead to mobbing. When produced together the production plausibly owes to one and the same situation, and mobbing may be the appropriate response to a situation both licensing alert (danger) and calling for recruitment.

While several of the proposed compositional calls in the literature are intriguingly similar to ABC + D (southern pied babbler mobbing calls (Engesser et al. 2016), chimpanzee alarm-hu + waa bark (Leroux et al. 2023); cf. Schlenker et al. 2022) it remains open that some complex calls are not amenable to such an analysis. Schlenker et al’s discussion brings out two broader points: (i) although compositionality presupposes syntax, the converse doesn’t hold; (ii) there is not a strong case for compositionality where the meaning of the whole amounts to the sum of the components (plus background knowledge), i.e. to what the components would convey together if each simply contributed its meaning separately (=“ABC and D”). Cases like the latter Schlenker et al. refer to as **trivial compositionality**, since syntax – the combining of the parts – need not contribute anything to yield the attested meaning of the whole.

Suzuki et al. (2017) already acknowledged another *non-compositional* alternative, one which may be more broadly applicable: that ABC + D is **directly associated** with its meaning as a unit (§ 2). For example, ABC + D may simply mean “mob” (Schlenker et al. 2016b, 2023; Suzuki et al. 2017). On the face of it this alternative theory may seem rooted in mere skepticism and less explanatory: after all, the meaning of the whole (“mob”) appears to be related to the meaning of the parts (“alert”, “recruit”) non-arbitrarily. But such whole-part relatedness is not unexpected *even if* the meaning is directly associated, given either of two plausible scenarios for how the relevant calls came to be: that the complex call emerged after its components, or the reverse, that the component calls were “backformed” from re-analysis of a simplex call into a complex one. As an example of the former, one possibility is that ABC and D, because they provide relevant information in situations calling for mobbing, tended to be produced in such situations. This in turn could have lead to fossilization of the calls into a *compound* mobbing call sans compositionality. Thus, to establish compositionality – whose emergence would itself call for explanation – it is not sufficient to simply identify a call with apparent parts whose meaning is related to theirs.

As discussed in § 2, compositionality gains explanatory power, and in turn plausibility, if there are *systematic* contributions of form (e.g. combination) to meaning. Berthet et al. (2025) identify four putatively compositional call combinations in bonobos, but no general rule is identified mapping the meanings of parts to wholes *across* combinations. (In addition, one of the four appears to be a case of trivial compositionality). If the meaning of each combination relates in an idiosyncratic way to its parts, other considerations are needed to support compositionality over the alternative. In the direction of systematicity, Girard-Buttoz et al. (2025) propose a method to identify broad types of part-whole meaning dependencies. This is done by comparing the frequency distributions of situations of use for call combinations (P + Q) vs. their components (P, Q). In a study of chimpanzee call combinations they identify two dependencies which could indicate use of compositional rules: (i) P + Q is used in a subset of that situations P that is (alt. Q) (“modification of P by Q” (or vice versa) (ii) P + Q is used in the intersection of the set of situations P and Q are used in.^4^ However, for each of these dependencies only one call combination is indentified that unambiguously fits it. Several others seem to instantiate (i) in multiple ways (P modifies Q and Q modifies P) or both (i) and (ii), which remains unexplained. In addition, (ii) is the signature of trivial compositionality. Thus, while these two studies provide data relevant to establishing systematicity, future research will require additional, finer-grained analyses to establish the nature and extent of form-meaning correspondences across calls. The number of complex calls attested, which thus far appears small, may be a limiting factor to make a strong case for compositionality based on systematicity.

Even for a small (thus finite) system of calls, a strong argument for compositionality is possible from competence with novel expressions, if the system’s rules or simplex calls are learned or open-ended (§ 2). For example, although Suzuki et al. identified only one complex call produced by Japanese great tits, the ABC + D mobbing call, they devised an experiment to test compositionality against direct association. An artificial hence novel call was constructed by replacing D with the “synonymous” tää recruit call of willow tits, a related, sympatric species, to which Japanese tits respond as if it were their own D call. Japanese tits were shown to respond to playback of ABC + tää in a similar way to their own ABC + D mobbing calls. This was taken to support use of a compositional rule, mapping an ordered combination “alert”+“recruit” to “mob” (see Schlenker et al. (2024) for a concrete proposal about this mapping).

This conclusion rests on the assumption that responses to ABC + tää depended on tää being learned through cohabitation in mixed flocks with willow tits, and thus that the Japanese tit call system is open-ended, at least with respect to comprehension. While there is evidence that many unrelated species come to understand the content of each other’s calls through experience, there is also evidence, from another subspecies of great tits, that experience is not always necessary: European great tits respond to the mobbing calls of a related but *allopatric* species, black-cap chickadees (Dutour et al. 2017; Randler 2012; Salis et al. 2020). This raises the possibility that responses to ABC + tää were not dependent on learning, but rather due to the kind of mechanism underlying allopatric mobbing call recognition in tits, which remains consistent with direct association. A possible such mechanism is shared acoustic properties (Randler 2012; Salis et al. 2024), e.g. a conserved prototype. While Suzuki et al. included a control intended to rule out “acoustic similarity” to ABC + D as an alternative explanation to compositionality – a manipulation of tää, shortened to make it closer in duration to D, there are least two open issues.

First, the logic is that the manipulation increased similarity to D, and thus that the observed failure to respond to the controls refutes acoustic similarity. However, the manipulation may have (also) induced something unnatural sounding to the birds or obscured the properties necessary to drive response. Further research identifying these properties could allow for a clearer control. Second, only results for playback of shortened tää *on its own* are reported, not for playback of ABC + shortened tää, which is the more direct test. This leaves open the possibility that responses to ABC+(shortened) tää are driven by the mechanism underlying allopatric call recognition, e.g. a conserved mobbing call prototype, even if responses to tää alone are not. Clearer support for Suzuki et al.’s conclusion could come from demonstrating non-response to ABC + tää by Japanese tits that have not previously cohabited with willow tits.

## Conclusion

The compositionality of human language is evident in systematic connections between form and meaning. Strong support for compositionality can be given by appeal to further properties of human languages which guarantee the possibility of novel calls: learning, open-endedness and recursion. Potential differences in these respects, as well as in size and extent, complicate the arguments made thus far for compositionality in animal communication systems. Future research should aim to (i) identify regular contributions of form to meaning *across* calls with the same structure; (ii) establish whether the systems have properties that guarantee novel calls, and whether the conditions to test them are accessible. Ultimately further considerations may be relevant or necessary, e.g. theory internal ones; cf. Schlenker et al. (2014). In any case, the systematic and comparative study of complex signals in other species is valuable for understanding the evolution of these systems (Schlenker et al. 2022), and ultimately for situating compositionality in the evolution of human language.

## Data Availability

No datasets were generated or analysed during the current study.

## Footnotes

1 For background on compositionality and other linguistic concepts in the context of research on animal communication see Berthet et al. (2023). For a general introduction to linguistic concepts used in this article see e.g. Fromkin et al. (2019).

2 A *possible* example comes from Japanese great tits. While there is evidence that their natural call system is innate (e.g. Suzuki (2011), there may also be evidence that it can be extended through learning in the right circumstances (Suzuki et al. 2017). Cf. § 3.

3 Here and throughout ‘meaning’ is intended in the neutral sense of information carrying, following the works under discussion. The question of whether animal calls encode information compositionally is in principle independent of comparative questions about whether they are symbolic in nature and (related) questions about the cognitive mechanisms underlying their use (e.g. whether they are produced intentionally to convey information to others).

4 The vocal repertoire identified by Girard-Buttoz et al. may be more liberal than prior literature, in identifying some calls as combinations that others assume to be simplex (syntactically unstructured); cf. Leroux 2023. For present purposes, it suffices to note that failure to demonstrate systematicity in a *larger* repertoire of combinations would underscore the points made above. All things equal, a larger repertoire provides more opportunity for systematicity.
